# Bioinformatics Analysis of *Cereus*-Derived Peptides Targeting β‑Lactamases and Bilayer Membrane from *Klebsiella pneumoniae* and *Acinetobacter baumannii*

**DOI:** 10.1021/acsomega.5c13072

**Published:** 2026-06-02

**Authors:** João A. Teodoro, Maria Izadora O. Cardoso, Graziela S. Virgens, Danilo T. Amaral

**Affiliations:** Centro de Ciências Naturais e Humanas, Universidade Federal do ABC (UFABC), Santo André 09210-170, São Paulo, Brazil

## Abstract

Antimicrobial resistance (AMR) represents a major global public health challenge, arising from the ability of microorganisms such as bacteria, fungi, and viruses to develop mechanisms that render them insensitive to conventional antibiotics. A key factor contributing to this problem is the production of β-lactamase enzymes, which confer resistance to multiple antibiotic classes and are associated with multidrug-resistant pathogens such as *Pseudomonas aeruginosa*, *Acinetobacter baumannii*, and *Klebsiella pneumoniae*, responsible for severe and often fatal hospital-acquired infections. In addition, the phospholipid membrane model was generated using CHARMM-GUI to provide insight into peptide-membrane interactions and their potential to induce membrane disruption. In this context, developing new therapeutic strategies, such as antimicrobial peptides (AMPs), has emerged as a promising alternative capable of acting against resistant microorganisms. This study investigates, through *in silico* analyses using tools such as Fpocket, FTsite, FTmap, and HADDOCK, to evaluate the interactions between five AMPs derived from Neotropical *Cereus* (mandacaru) species against β-lactamases from *K. pneumoniae* and *A. baumannii*. As a result, all five *Cereus*-derived AMPs showed favorable predicted interactions with β-lactamase interaction sites, and CF267 and CJ149 exhibited stable engagement with the phospholipid bilayer, supporting their potential to disrupt membranes in the simulated system. Docking analyses indicated favorable affinities for all peptides, with CF267 × 3RXX and CJ149 × 3RXX emerging as the most promising complexes due to their highly negative energy scores. These findings suggest favorable predicted interactions with β-lactamase binding regions, indicating potential molecular compatibility against AMR mechanisms in *A. baumannii* and *K. pneumoniae*. Upcoming *in vitro* assays will be essential to validate these predictions.

## Graphical abstract

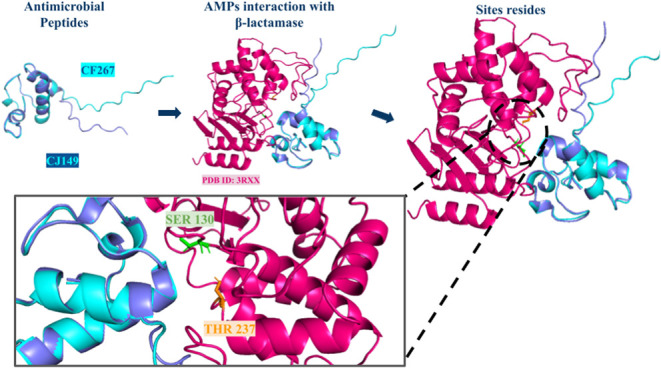

## Introduction

Antimicrobial resistance (AMR) arises when bacteria, fungi, viruses, and parasites develop mechanisms that allow them to withstand conventional antibiotics and other antimicrobial agents.^1^ Consequently, AMR has emerged as an urgent global public health challenge, making the search for novel antibacterial strategies indispensable. This necessity stems from the increasing difficulty in treating resistant infections and the escalating risk they pose.^1–3^ Projections indicate that, by 2050, antibiotic-resistant bacterial infections could account for up to 10 million deaths annually worldwide.^2^

A major factor contributing to antimicrobial resistance is the production of β-lactamase enzymes by certain microorganisms.^4^ These proteins confer resistance to several classes of traditional antibiotics and are strongly associated with multidrug-resistant (MDR) pathogens such as *Pseudomonas aeruginosa*, *Acinetobacter baumannii*, and *Klebsiella pneumoniae*.^5,6^ These pathogens are linked to high morbidity and mortality in healthcare-associated infections and remain a persistent challenge in clinical practice. The most critical group includes MDR bacteria that pose a particular threat in hospitals and nursing homes,^7^ requiring urgent action, especially in infections affecting immunocompromised individuals.^6^ Severe cases are frequently observed in elderly patients with *A. baumannii* infections and in individuals with serious war-related injuries. Resistance to conventional antibiotics, including penicillins, cephalosporins, and carbapenems, has increased significantly.^7,8^

In the case of *A. baumannii*, its clinical relevance is largely associated with its remarkable ability to persist in hospital environments, making it a particularly dangerous nosocomial pathogen. This microorganism serves as a persistent source of infection, remaining viable on surfaces for extended periods. Consequently, hospitalized patients are at high risk of acquiring MDR infections. Notably, some studies have reported that more than 48% of hospital surfaces may be contaminated with *A. baumannii*.^9^ Another important opportunistic human pathogen is *P. aeruginosa*, which can cause a wide range of infections, including respiratory tract infections, endocarditis, urinary tract infections, and septicemia. Owing to the difficulty in treating its resistance, *P. aeruginosa* is recognized as one of the six leading mortality-causing pathogens and was associated with 3.57 million deaths globally in 2019 due to AMR.^10^

To address AMR caused by diverse pathogenic microorganisms, one promising strategy is the development of novel therapeutic approaches. Among these, antimicrobial peptides (AMPs) have emerged as a compelling alternative.^1^ AMPs or host-defense peptides are short amino acid residues, typically ranging from 3 to 100 in length,^11–13^ and can be derived from a wide variety of sources, including plants, mammals, amphibians, insects, aquatic organisms, and microorganisms.^14–16^ Most AMPs are cationic, with a net charge ranging from +2 to +9, with hydrophobicity and amphipathicity properties.^11,14,17^ In plant species, AMPs generally have a molecular weight between 2 and 10 kDa and often contain 4 to 12 cysteine residues.^12,18^

Recent research has evaluated potential AMPs from the Neotropical Mandacaru (*Cereus*) species that may act against certain pathogens.^16^ To advance this work, further *in silico* studies were carried out to investigate the interactions between selected peptides (CF15, CF267, CH167, CH176, and CJ149) and two β-lactamase proteins from distinct microorganisms: *K. pneumoniae* (PDB ID: 3RXX) and *A. baumannii* (PDB ID: 4U0T). Computational approaches, including pocket and site prediction, molecular docking, and the CHARMM-GUI platform analysis. This study aims to investigate the interactions between β-lactamases from two pathogenic species and five AMPs, to explore their potential molecular interactions and possible relevance for the development of novel therapeutic agents against AMR. We hypothesize that the molecular interactions between these AMPs and β-lactamases may reveal promising insights for the development of new strategies to combat AMR and improve current treatment options.

## Materials and Methods

### Cereus Peptides

The peptides were selected based on previous studies from three *Cereus* species: *Cereus fernambucensis* (CF15 and CF267), *Cereus hildmannianus* (CH167 and CH176), and *Cereus jamacaru* (CJ149).^16^ All peptides are cationic with a positive charge greater than 5, have a molecular weight below 10 kDa, contain 8 to 12 cysteine residues, and range from 74 to 92 amino acids in length (Table 1). Previous analyses have indicated that these AMPs possess potential antimicrobial properties, including antibacterial and antifungal activity, and interact with membranes via an external mechanism. In the *in silico* analyses, none of the peptides exhibited potential allergenicity or toxicity toward eukaryotic cells.^16^

**Table 1:** Five *Cereus*-Derived AMPs Were Used in This Study, along with Their Corresponding Peptide, Specific Classes, and Physicochemical Properties, Such as Length, Charge, Molecular Weight, Isoelectric Point, and Hydrophobicity

peptide	protein class	species	length (aa)	charge	mol wt	pI (isoelectric point)	hydrophobicity
CF15	snakin	*C. fernambucensis*	84	11.34	9211.69	9.3	–0.79
CF267	snakin	*C. fernambucensis*	78	7.25	8601.87	8.69	–0.77
CJ149	snakin	*C. jamacaru*	74	7.25	8241.51	8.69	–0.84
CH176	defensin	*C. hildmannianus*	92	5.5	9049.58	8.77	0.30
CH167	snakin	*C. hildmannianus*	76	9.25	7996.34	8.99	–0.36

### β-Lactamase

The β-lactamase proteins from Gram-negative bacteria were selected from two pathogenic species, *K. pneumoniae* (PDB ID: 3RXX) and *A. baumannii* (PDB ID: 4U0T). These proteins were chosen based on previous studies highlighting their role in MDR hospital infections,^5,7^ and were retrieved from the RCSB Protein Data Bank (https://www.rcsb.org). Both structures have a resolution better than 2 Å, indicating acceptable structural quality. And the PDB structures were cleaned and filtered to retain only one chain, enabling accurate preparation for the subsequent computational procedures.

### Pockets and Binding Interactions

The identification of potential binding pockets in the selected *Cereus* peptides was performed to explore structural features that may contribute to their antimicrobial activity and reveal sites potentially suitable for ligand recognition or molecular modulation. FPocket 1.0.1 (https://durrantlab.pitt.edu/fpocketweb/)^19^ web server was employed to detect structural cavities based on geometric and physicochemical descriptors, including pocket volume, depth, hydrophobicity, and drugability, thereby allowing the characterization of accessible binding regions. This approach enabled the characterization of solvent-accessible binding regions potentially involved in molecular interactions relevant to peptide functionality.

To further characterize binding hot spots, FTMap (https://ftmap.bu.edu/)^20^ was employed. This computational fragment-mapping method mimics experimental fragment screening by distributing a library of 16 small organic probe molecules (Table S1, showing main features of the probes used in FTSite and FTMap). FTMap identifies energetically favorable binding positions for each probe type, clusters the probes based on spatial and energetic proximity, and defines consensus sites (CSs) as regions where multiple probe clusters overlap. Complementary predictions of potential ligand-binding residues were performed using FTSite (https://ftsite.bu.edu/).^21^ FTSite identifies energetically favorable ligand-binding sites by combining geometric analysis with fragment-based energy mapping, using a reduced set of probe molecules and a scoring function optimized for protein–ligand recognition.

### Molecular Docking

The High Ambiguity Driven protein–protein Docking (HADDOCK) v. 2.4 web server (https://rascar.science.uu.nl/haddock2.4) was applied to evaluate the potential interactions of our biomolecular complexes.^22,23^

In this step, the β-lactamase PDB structures and the peptide PDB files were uploaded to simulate their interactions and potential binding affinities. Ten docking analyses were performed, testing five peptides against two target proteins. The resulting scores were evaluated, and the docking clusters were examined to determine whether interactions occurred in the pocket region, identifying the amino acid residues involved in the strongest and most favorable binding. Additional parameters, such as van der Waals, electrostatic, and desolvation energies, were also analyzed to characterize the nature and stability of the predicted complexes.^23,24^ This analysis provided insights into the potential mechanisms of action of these peptides against MDR infections.

### Charmm GUI

To reproduce the physicochemical environment of a Gram-negative bacterial membrane and evaluate the interaction of *Cereus* AMPs with it, we employed CHARMM-GUI, a web-based platform for molecular mechanics and dynamics simulations.^25^ Specifically, the Membrane Builder module (http://www.charmm-gui.org/input/membrane) was used to construct all-atom lipid bilayer systems mimicking the *Campylobacter jejuni* membrane.

Following the standard workflow provided by CHARMM-GUI, a bilayer composed of POPE:POPG (8:2) lipids was generated and complemented with lipopolysaccharides (LPS) to reproduce the outer leaflet composition typical of Gram-negative bacteria. The system was solvated with a 40 Å water layer and neutralized by adding counterions. Protein orientation was validated along the *Z*-axis, ensuring the bilayer center was aligned at *Z* = 0 before energy minimization and equilibration.

Subsequently, the AMP structures (PDB format) were uploaded into the CHARMM-GUI interface to model their *in silico* interactions with the bacterial membrane under controlled physicochemical conditions. All parameters were configured according to the official CHARMM-GUI tutorials and previous methodological references.^25,26^

## Results and Discussion

To ensure clarity, our results are organized according to the main analytical stages of the *in silico* pipeline: (i) selection of the top-performing AMPs from previous studies,^16^ (ii) characterization and identification of β-lactamase binding pockets and active sites, (iii) molecular docking analyses, and (iv) simulation of AMP interactions with the phospholipid bilayer membranes of Gram-negative pathogens.

### Selection of AMPs and β-Lactamase

Peptides were selected from Teodoro et al.^16^ based on: (i) the highest predicted antimicrobial probability scores as antibacterial (ABPs) and antifungal proteins (AFPs) with strong predicted interactions against pathogen proteins; (ii) favorable physicochemical parameters (net charge, hydrophobicity, stability); and (iii) absence of predicted toxicity. These criteria guided the prioritization of the five candidates evaluated here (CF15, CF267, CH167, CH176, and CJ149). We evaluated the *Cereus* peptides interaction to β-lactamase protein from two pathogenic species, *K. pneumoniae* (PDB ID: 3RXX; 1.62 Å resolution; 264 amino acids) and *A. baumannii* (PDB ID: 4U0T; 1.73 Å resolution; 360 amino acids), and the bilayer membranes from Gram-negative species.

The physicochemical properties of the five *Cereus*-derived peptides were analyzed to better understand their stability, hydrophobicity, and folding patterns related to antimicrobial activity (Table 2). All peptides were classified as *unstable* according to the instability index (>40), a feature frequently associated with bioactive peptides that require conformational flexibility for interaction with membranes and enzymatic targets.^27,28^ The aliphatic index varied markedly between peptides, ranging from 22.43 (CJ149) to 75.43 (CH176), suggesting differences in thermostability and side-chain hydrophobic packing.^29^ The GRAVY values were predominantly negative (−0.79 to −0.36), confirming the hydrophilic and amphipathic nature of these molecules, consistent with antimicrobial peptides that insert partially into lipid bilayers.^30^ Estimated half-lives ranged from 0.8 h in mammalian reticulocytes to >10 h in *Escherichia coli*, indicating short cytoplasmic persistence but potential stability in bacterial systems. This feature may favor transient but potent bioactivity upon secretion or heterologous expression.^31^

**Table 2:** Physicochemical Characterization of the Five *Cereus*-Derived Peptides

peptide	CF15	CF267	CH167	CH176	CJ149
extinction coefficients	Extinction coefficients are in units of M^–1^ cm^–1^, at 280 nm measured in water.	Extinction coefficients are in units of M^–1^ cm^–1^, at 280 nm measured in water.	This protein does not contain any Trp residues. Experience shows that this could result in more than 10% error in the computed extinction coefficient.	This protein does not contain any Trp residues. Experience shows that this could result in more than 10% error in the computed extinction coefficient.	Extinction coefficients are in units of M^–1^ cm^–1^, at 280 nm measured in water.
			Extinction coefficients are in units of M^–1^ cm^–1^, at 280 nm measured in water.	Extinction coefficients are in units of M^–1^ cm^–1^, at 280 nm measured in water.	
Ext. coefficient	12,210	17,710	3730	4970	17,710
Abs0.1% (=1 g/L)	1.325	2.059	0.466	0.549	2.149
Ext. coefficient	11,460	16,960	2980	4470	16,960
Abs0.1% (=1 g/L)	1.244	1.972	0.373	0.494	2.058
estimated half-life	The N-terminal of the sequence considered is Q (Gln).	The N-terminal of the sequence considered is Q (Gln).	The N-terminal of the sequence considered is D (Asp).	The N-terminal of the sequence considered is A (Ala).	The N-terminal of the sequence considered is Q (Gln).
the estimated half-life is	0.8 h (mammalian reticulocytes, *in vitro*).	0.8 h (mammalian reticulocytes, *in vitro*).	1.1 h (mammalian reticulocytes, *in vitro*).	4.4 h (mammalian reticulocytes, *in vitro*).	0.8 h (mammalian reticulocytes, *in vitro*).
	10 min (yeast, *in vivo*).	10 min (yeast, *in vivo*).	3 min (yeast, *in vivo*).	>20 h (yeast, *in vivo*).	10 min (yeast, *in vivo*).
	10 h (*E. coli* *, in vivo*).	10 h (*E. coli* *, in vivo*).	>10 h (*E. coli*, *in vivo*).	>10 h (*E. coli* *, in vivo*).	10 h (*E. coli* *, in vivo*).
instability index	47.73	69.30	50.57	41.34	63.96
aliphatic index	25.60	25.00	53.95	75.43	22.43
grand average of hydropathicity (GRAVY)	–0.793	–0.771	–0.361	0.299	–0.836
PS	This classifies the protein as unstable.	This classifies the protein as unstable.	This classifies the protein as unstable.	This classifies the protein as unstable.	This classifies the protein as unstable.

Structural models (Table 3) revealed that all peptides adopt compact conformations dominated by short α-helices and flexible coil regions. Among them, CF267 and CJ149 presented the highest solvent-accessible surface areas (SASA: 6385.2 Å^2^ and 5684.4 Å^2^) and contact densities (≈8.8), with five disulfide pairs contributing to structural stabilization. These characteristics indicate high conformational adaptability, typical of amphiphilic AMPs capable of both binding enzymatic clefts and permeabilizing membranes.^32,33^

**Table 3:** Computed Structural Features of the Peptide Models

peptide	SASA total	fraction hydrophobic surface	RG	disulfide pairs	PLDDT mean	PLDDT median	fraction PLDDT LT70	contact density
CF15	6.437.439.939.297.760	1.893.935.395.891.290	14.262.396.812.438.900	4	8.962.168.503.937.000	96.94	13.070.866.141.732.200	880.952.380.952.381
CF267	6.385.253.934.758.260	2.564.583.345.244.110	16.833.723.068.237.300	5	9.045.354.729.729.720	96.91	16.722.972.972.972.900	79.743.589.743.589.700
CH167	6.102.937.534.178.350	2.802.043.956.506.300	14.054.791.450.500.400	5	8.640.786.106.032.900	93.94	1.882.998.171.846.430	8.078.947.368.421.050
CH176	4.948.629.155.325.500	29.029.237.540.362.900	11.739.800.453.186.000	3	9.584.223.642.172.520	96.44	0.0	9.0
CJ149	5.684.448.604.052.580	25.136.853.615.335.500	13.379.679.679.870.600	5	8.589.365.079.365.070	93.44	1.746.031.746.031.740	8.621.621.621.621.620

Specifically, CF267 and CJ149 exhibited an optimal balance between hydrophilicity and surface exposure, which may facilitate hydrogen-bonding and electrostatic interactions with the catalytic residues of β-lactamases, such as Ser130 and Thr237 in *K. pneumoniae* and Ser64 and Ser315 in *A. baumannii*, as observed in the subsequent docking results. Their hydrophilic character (GRAVY −0.77 to −0.84) aligns with the observed docking stability and complements the negative GRAVY of many plant AMPs known to disrupt Gram-negative membranes.^34^

These physicochemical and structural descriptors are consistent with a possible dual mechanism of action for *Cereus*-derived AMPs, including (i) predicted interaction with β-lactamase enzymes binding regions, as suggested by the hydrogen-bonding patterns in docking simulations, and (ii) potential disruption of bacterial membranes through amphipathic, α-helical motifs. This is consistent with the multifunctionality reported for cationic AMPs from other plant sources, such as *Capsicum annuum*.^35^

### Pockets and Binding of β-Lactamase

To characterize the β-lactamase structures of these two species, we employed three computational tools: Fpocket, FTSite, and FTMap. The initial analysis performed in the Fpocket server identified 15 potential pocket regions for *K. pneumoniae* (Table S2 shows PDB IDs of the two β-lactamase types and their corresponding binding pockets), with pocket #5 showing the highest druggability score (0.424). For *A. baumannii*, 19 potential pockets were detected, and pocket #1 and #6 exhibited the best druggability score (0.855 and 0.541, respectively) (Table S2). These results indicate that the identified pockets represent potential binding sites for ligands or drugs capable of inhibiting enzymatic activity and modulating resistance phenotypes. Druggability scores range from 0 to 1, with values greater than 0.5 considered indicative of druggable sites.^36^

Additional analyses were performed to refine and confirm these initial findings. To this end, the FTSite server analysis revealed three promising binding regions, which could potentially interact with various compounds and serve as targets for therapeutic intervention (Table S3 shows the association between the β-lactamase structures and the structural sites that correspond to the predicted potential pocket regions). These regions are crucial for understanding how ligands bind to proteins and which amino acid residues are involved in hydrogen bonding, salt-bridge formation, and other molecular interactions. Notably, the locations of these binding sites differed between the two pathogen-derived β-lactamases. This occurs because, despite belonging to the same protein class, they exhibit specific variations in their amino acid sequences (Figure 1). When comparing the pocket regions with the binding sites identified by FTSite, several areas of overlap were observed. Based on the previous Fpocket results, in the β-lactamase from *K. pneumoniae* (PDB ID: 3RXX), site #1 corresponded to pockets #2, #4, and #9; site #2 to pocket #5; and site #3 to pocket #1. In the β-lactamase from *A. baumannii* (PDB ID: 4U0T), site #1 matched pockets #4, #7, and #10; site #2 with pocket #10; and site #3 with pockets #6 and #17 (Table 4 and Figure 2). These findings highlight the importance of integrating results from multiple web servers to obtain complementary information. In this context, for the first protein, the pocket with the highest druggability score (pocket #5) corresponds to site #2. For the second protein, the most relevant pockets are #1 and #6, where pocket #6 overlaps with site #3, and pocket #1 lies close to sites #1 and #2 (Figure 2). This comparison provides an important foundation for the next stage of analysis, which aims to validate *in vitro* these findings and identify new potential binding opportunities. It is important to emphasize that regions with favorable interactions are not always limited to those with the highest druggability scores, since effective ligand binding can occur in alternative sites with strong interaction potential.

**Figure 1:**
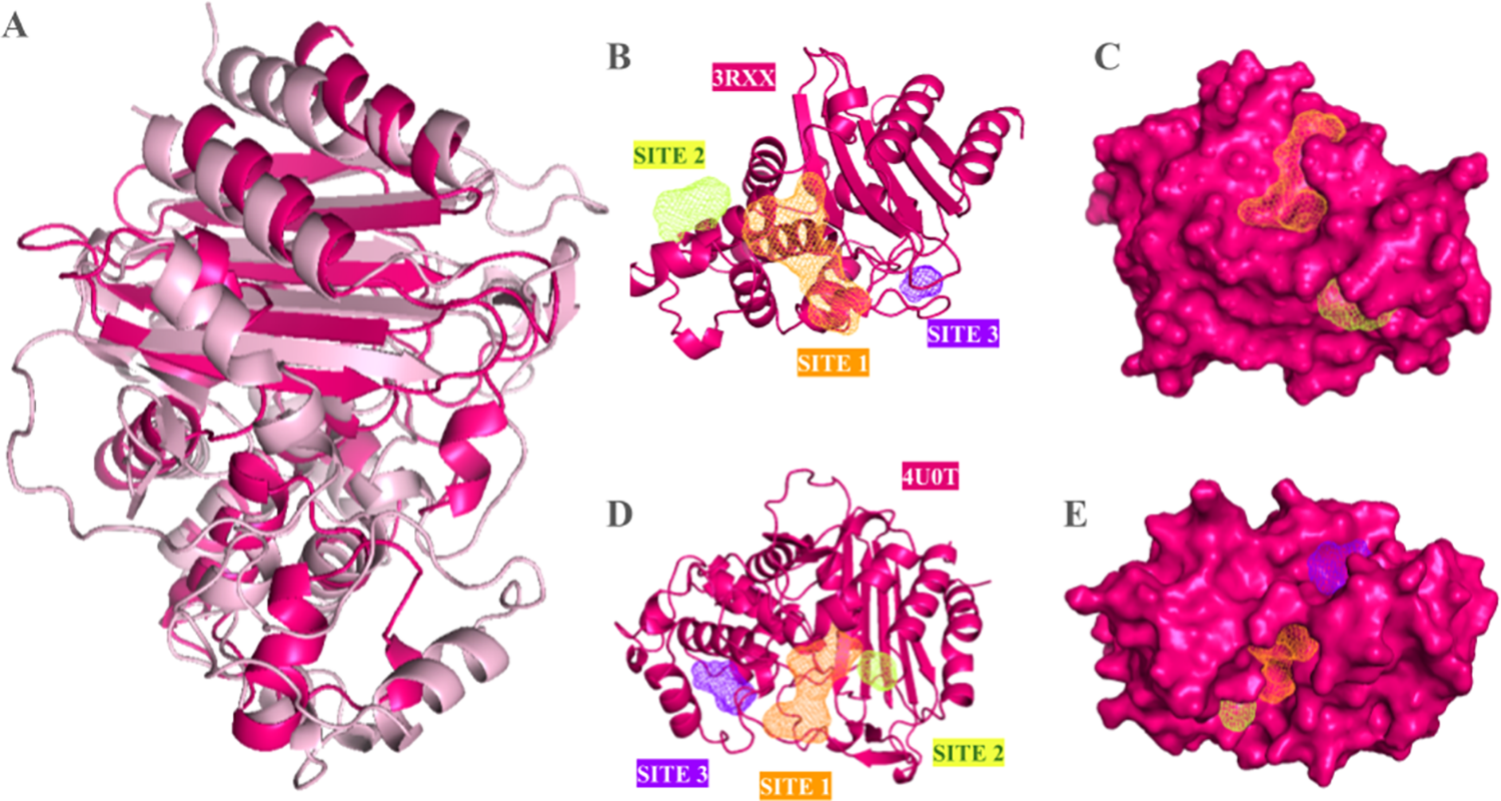
(A) The β-lactamase from *K. pneumoniae* (PDB ID: 3RXX, shown in magenta) and the β-lactamase from *A. baumannii* (PDB ID: 4U0T, shown in light pink) are superimposed, highlighting their structural correlation and the differences among specific residues that may indicate variations in their potential interaction regions and binding sites. (B) β-lactamase from *K. pneumoniae* (PDB ID: 3RXX) showing three potential interaction site regions: site #1 (orange), site #2 (green), and site #3 (purple). (C) Cavities of *K. pneumoniae* protein corresponding to these three sites, highlighting that all of them exhibit noticeable depth and pocket-like features. (D) β-lactamase from *A. baumannii* (PDB ID: 4U0T) showing three potential interaction site regions: site #1 (orange), site #2 (green), and site #3 (purple). (D) Cavities of *A. baumannii* protein corresponding to these three sites, highlighting that all of them exhibit noticeable depth and pocket-like features.

**Figure 2:**
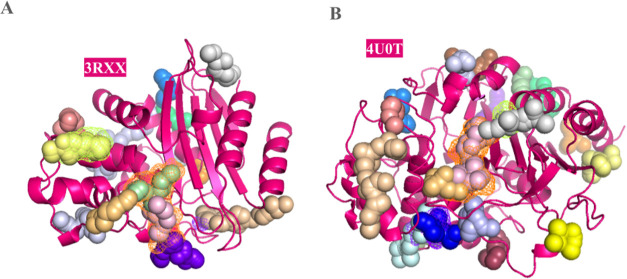
Predicted binding site regions and potential druggable areas identified by FTSite. (A) Fifteen potential druggable regions in *K. pneumoniae* β-lactamase (PDB ID: 3RXX). (B) Nineteen potential druggable regions in *A. baumannii* β-lactamase (PDB ID: 4U0T).

**Table 4:** Interactions between β-Lactamases from *K. pneumoniae* and *A. baumannii* and Antimicrobial Peptides (AMPs), Showing the Interacting Residues, Their Corresponding Binding Sites, and the Number of Interactions Per Site^a^

peptide	β-lactamase	site #1	residue (*n*°)	residue	site #2	residue (*n*°)	residue
CJ149	4u0t	SER315	4	Ala11, Pro12, Ser13, Gly14	SER64	-	-
CJ149	3rxx	SER130	3	Arg32, Tyr36, Asn68	THR237	1	Asn68
CH176	4u0t	SER315	-	-	SER64	-	-
CH176	3rxx	SER130	6	Cys4, Gly5, Ala6, Ala8, Lys9, Thr12	THR237	2	Lys9, Thr12
CH167	4u0t	SER315	2	Arg27 e Leu28	SER64	-	-
CH167	3rxx	SER130	3	Arg27, Leu28, Thr53	THR237	1	Arg27
CF267	4u0t	SER315	1	Arg49	SER64	-	-
CF267	3rxx	SER130	3	Arg47, Cys48, Arg49	THR237	2	Arg47, Arg49
CF15	4u0t	SER315	-	-	SER64	-	-
CF15	3rxx	SER130	4	Lys49, Ala52, Lys53, Trp74	THR237	4	Lys49, Ala52, Lys53, Trp74

a Some AMP residues share the same binding region.

To refine these findings, it is essential to identify which amino acid residues form hydrogen bonds (h-bonds) with a 5 Å distance, and whether they are located within the same regions as the previously identified pockets and binding sites. For the β-lactamase from *K. pneumoniae* (PDB ID: 3RXX), two key amino acid residues, Ser130 (orange) and Thr237 (green), were identified as forming the most hydrogen bond interactions. Ser130, located in site #1, interacted with 3–6 amino acid residues of the peptide ligands. In site #2, Thr237 established at least one hydrogen bond, with variable interaction numbers depending on the AMP: CH167 one interaction, CH176 and CF267 formed two, and CF15 formed four (Table 4). Considering both sites, CH176 and CF15 showed the highest overall number of hydrogen bond interactions with *K. pneumoniae* (eight total) (Chart 1), followed by CH167 (six), CF267 (five), and CJ149 (four) (Chart 1). In the case of *A. baumannii* (PDB ID: 4U0T), two key amino acid residues were identified: Ser315 (site #1 orange) and Ser64 (site #2 green). However, only CJ149, CH167, and CF267 displayed hydrogen bond interactions with residues from site #1, ranging from one to three interactions: CJ149 (four - Ala11, Pro12, Ser 13 and Gly14), CH167 (two - Arg27, Leu28), and CF267 (one - Arg49). None of the AMPs exhibited hydrogen bond formation with site #2 within the 5 Å cutoff, and the remaining peptides only engaged residues outside the predefined catalytic sites (Table 4).

**Chart 1:**
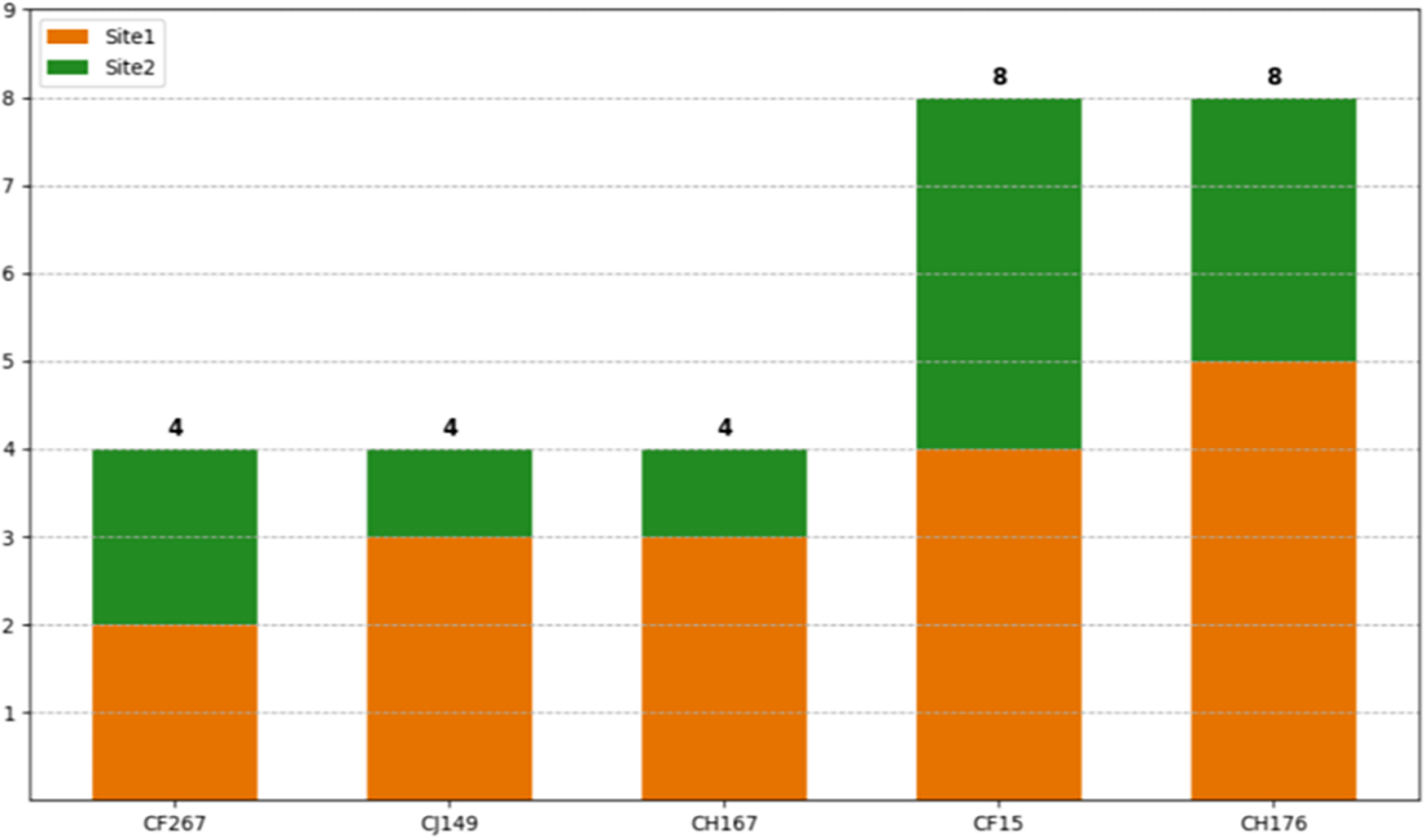
Graph Shows the Interactions between AMP Residues and Those of the Target Protein (PDB ID: 3RXX), with Site #1 Highlighted in Orange and Site #2 in Green[Fn c1fn1]

a These results represent the combined interaction data, although some AMP residues may interact with more than one site. This chart was generated by the VisDecode web server, available at https://visdecode.ai/.

The composition of antimicrobial peptides is closely associated with their ability to inhibit or inactivate a wide range of pathogenic microorganisms. The amino acids most commonly linked to this antimicrobial activity are those that appear in sequences enriched with one or more of the following residues: arginine (Arg), cysteine (Cys), glycine (Gly), histidine (His), lysine (Lys), and proline (Pro).^14,15^ In this context, the *A. baumannii* β-lactamase (PDB ID: 4U0T) showed interactions with *Cereus*-derived peptides containing one or more of these residues: CJ149 (Pro12, Gly14 at site #1), CH167 (Arg27 at site #1), and CF267 (Arg49 at site #1). Similarly, the *K. pneumoniae* β-lactamase (PDB ID: 3RXX) interacted with AMPs such as CJ149 (Arg32, Tyr36 and Asn at site #1, with Asn68 shared with site #2), CH167 (Arg27 shared between sites #1 and #2), CF267 (Arg47, Cys48, Arg49 and at site #1; Arg47 and Arg49 also shared with site #2), and CH176 (Cys4, Gly5, and Lys9 at site #1; Lys9, shared with site# 1 at site #2) (Table 4 and Chart 1). Based on these results, CF267 and CH176 exhibited the most favorable amino acid interactions with β-lactamases from both species, highlighting their potential as promising AMP candidates for further investigation.

#### Target and Ligand Interactions

To enhance these results, the potential functional and catalytic residues of the two β-lactamases, referenced in Table 4, are illustrated in Figure 3A, showing the key active-site residues. The region of this site is conserved between the two species, indicating a potentially strong interaction with AMPs that could bind and act against this resistance. This highlights the importance of selecting not only the best HADDOCK score but also the appropriate interaction region and residues. In this case, Ser130 and Thr237 in *K. pneumoniae* and Ser64 and Ser315 in *A. baumannii* exhibited strong interactions, with high numbers of hydrogen-bond interactions in the best-performing complexes CH176, CF15, CF267, and CH167 against β-lactamase (3RXX; Table 4).

**Figure 3:**
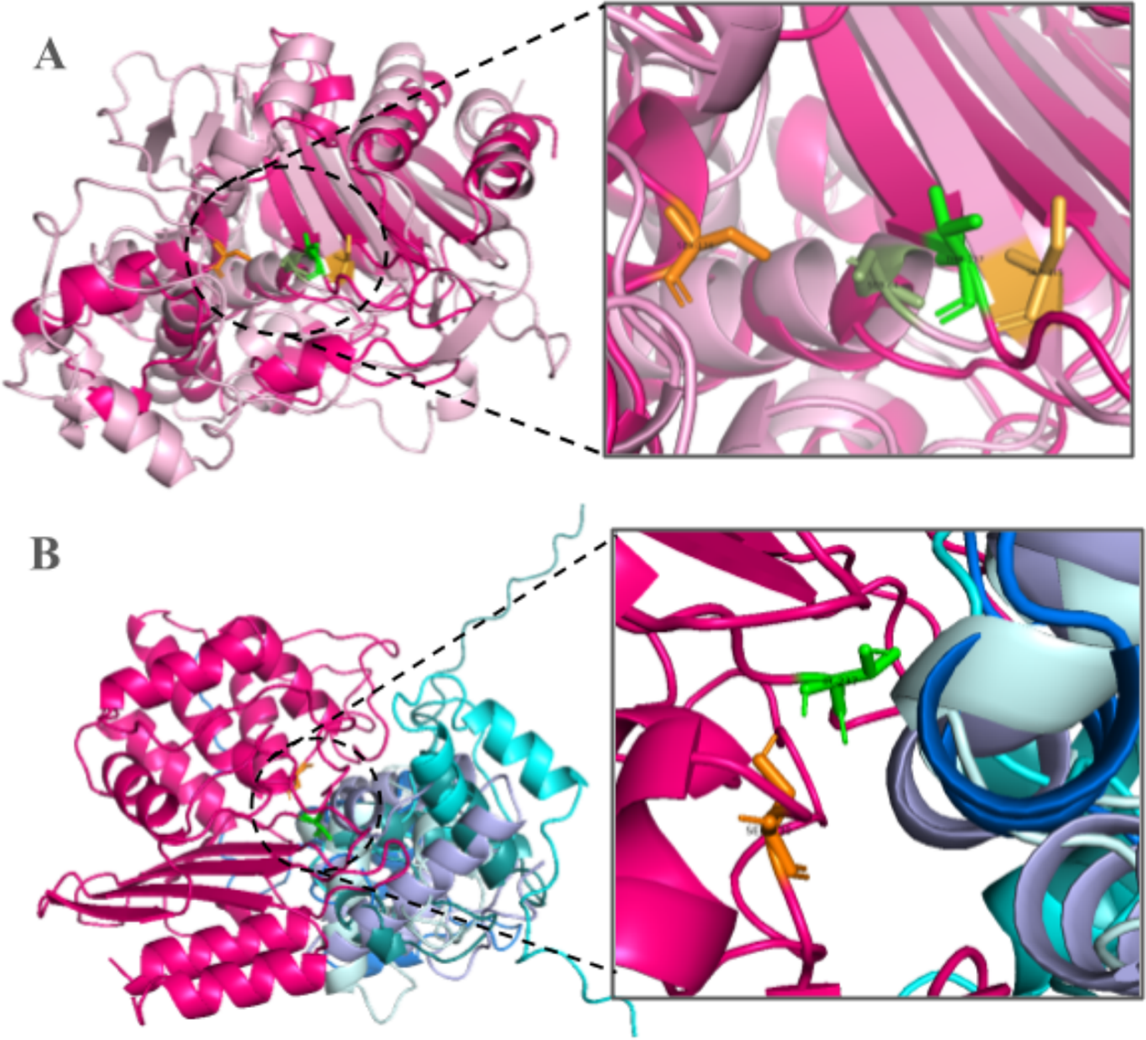
(A) Superposition of β-lactamases from *K. pneumoniae* (PDB ID: 3RXX, magenta) and *A. baumannii* (PDB ID: 4U0T, light pink), highlighting key catalytic residues: Thr237 (green) and Ser130 (orange) in *K. pneumoniae*, and Ser315 (bright orange) and Ser64 (smudge) in *A. baumannii*. A zoomed-in view emphasizes these residues. (B) Structural representation of *K. pneumoniae* (PDB ID: 3RXX, magenta) complexed with AMPs shown in various shades of blue: CJ149 (cyan), CH176 (light blue), CH167 (deep teal), CF267 (blue), and CF15 (light cyan). All peptides are positioned near the key amino acid residues Ser130 (orange) and Thr237 (green). A zoomed-in view emphasizes these two residues: Thr237 (green) and Ser130 (orange).

Based on these previous results, we performed molecular docking analyses to more precisely evaluate the interactions between the AMPs and the target pathogen proteins. The docking was carried out between β-lactamase enzymes and *Cereus*-derived peptides to improve upon the previous approaches. As shown in Table 5, some complexes exhibited considerably less favorable HADDOCK scores and weaker interaction networks, suggesting a lower likelihood of stable binding under physiological conditions.

**Table 5:** Docking Scores Obtained from the HADDOCK Server, Showing the Interaction Scores with Their Ranges, Van der Waals, Electrostatic, Desolvation Energy, and the RMSD from the Overall Lowest-Energy Structure for the Binding between Five AMPs and Two Species of β-Lactamases

peptide	β-lactamase	score	van der Waals energy	electrostatic energy	desolvation energy	RMSD (±)
CJ149	4u0t	181.2 ± 23.3	–76.6	–336.6	–6.5	12.3
CJ149	3rxx	60.9 ± 18.9	–68.5	–320.9	–7.3	12.4
CH176	4u0t	-	-	-	-	-
CH176	3rxx	202.0 ± 14.6	–62.8	–284.5	–26.9	0.4
CH167	4u0t	199.1 ± 16.0	–69.1	–202.6	–8.1	21.3
CH167	3rxx	126.5 ± 23.2	–61.1	–243.1	–11.3	13.8
CF267	4u0t	113.7 ± 10.8	–66.3	–188.8	–8.2	13.5
CF267	3rxx	43.1 ± 19.8	–72.4	–165.9	–22.9	8.8
CF15	4u0t	-	-	-	-	-
CF15	3rxx	153.5 ± 27.8	–70.3	–317.9	–16.9	0.7

For instance, CH176 and CF15 showed no hydrogen-bond interactions with residues Ser315 or Ser64 in *A. baumannii* β-lactamase (PDB ID: 4U0T). In contrast, CJ149 formed four hydrogen bonds involving Ala11, Pro12, Ser13, and Gly14; CH167 formed two involving Arg27 and Leu28; and CF267 formed one involving Arg49. All observed interactions occurred near site #1 (Ser315), while none of the peptides displayed interactions at site #2 (Ser64) (Table 4 and Figure 4). The corresponding function scores were 113.7, 181.2, and 199.1 for CJ149, CH167, and CF267, respectively. These findings suggest that among the analyzed peptides, CJ149 exhibits the most favorable binding properties, suggesting a potential interaction with the β-lactamase-mediated resistance mechanism in this Gram-negative pathogen.

**Figure 4:**
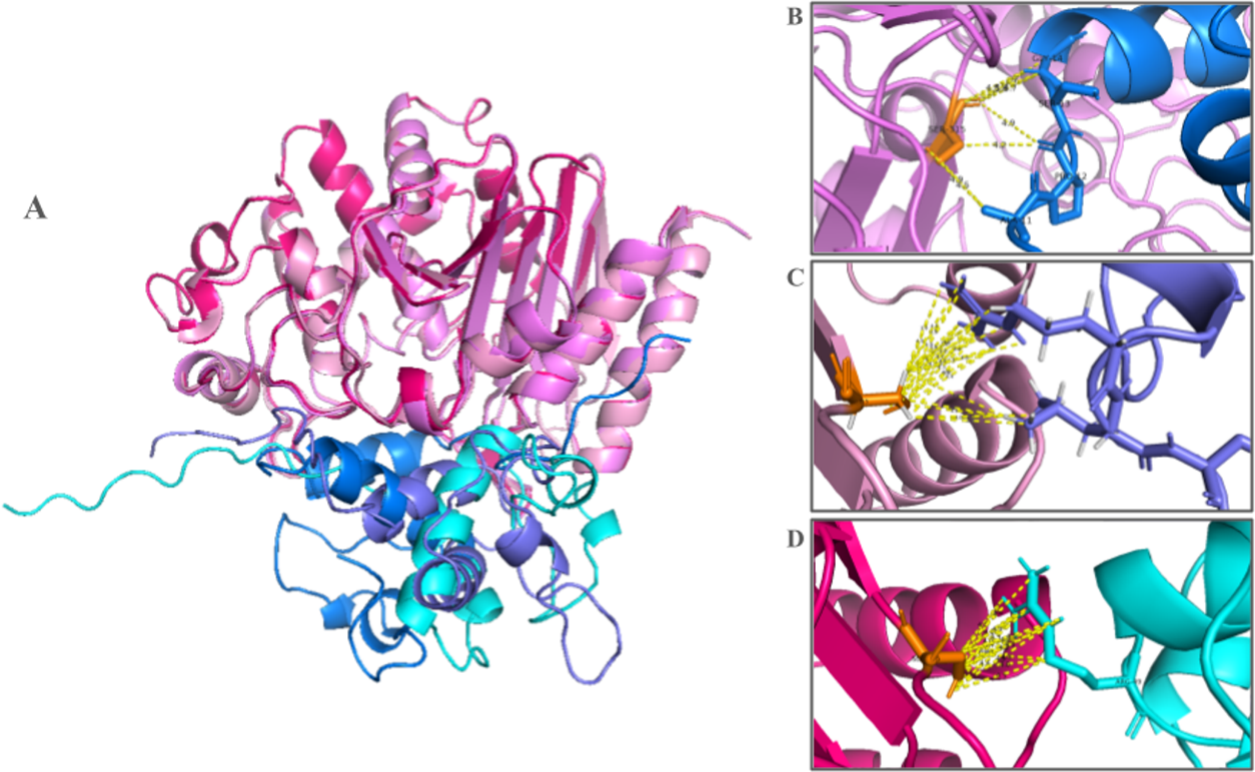
(A) Structural representation of *A. baumannii* (4U0T) shown in magenta, with the three AMPs represented in different shades of blue, corresponding to the complexes shown in panels (B–D). (B) β-lactamase from *A. baumannii* complexed with AMP CJ149. (C) β-lactamase from *A. baumannii* complexed with AMP CH167. (D) β-lactamase from *A. baumannii* complexed with AMP CF267. All complexes display hydrogen bonds with distances shorter than 5 Å.

For *K. pneumoniae* (PDB ID: 3RXX), all peptides interacted with both key residues, Ser130 (site #1, orange) and Thr237 (site #2, green), with interaction scores ranging from 43.1 to 202.0 (Table 5). As shown in Figure 3B, the AMPs CJ149, CH176, CH167, CF267, and CF15 are positioned close to the residues of the target protein. These peptide residues were previously identified in the FTMap analysis, further supporting the observed interactions. Specifically, CJ149 forms five hydrogen bonds with the target (four at site #1 and one at site #2); CH176 exhibits the strongest binding, with nine interactions (six at site #1 and two at site #2); CF267 form six interactions each (four at site #1 and two at site #2); CF15 shows eight interactions (four at site #1 and four at site #2) and CH167 shows four interactions (three at site #1 and one at site #2). These results suggest consistent hydrogen-bonding networks within the docking models that may contribute to the predicted stability of these complexes (Table 4 and Chart 1).

Using 202.0 as the highest (worst) score from CH176, consequently CF15 (153.5 ± 27.8) and 126.5 as the midpoint (calculated with the median of these value scores), the best interactions were observed for CF267 × 3RXX (43.1 ± 19.8), CJ149 × 3RXX (60.9 ± 18.9), and CH167 (126.5 ± 23.2). Furthermore, when correlating the lowest energy scores with the number of peptide residues interacting with the catalytic site, additional promising candidates emerge, particularly CF267 and CJ149, each showing interactions involving more than four residues. Notably, in CF267, residues Arg47 and Arg49 interact with both key residues of the β-lactamase, while in CJ149, residue Asn68 also establishes contacts with both catalytic residues (Tables 4 and 5; and Figure 5). Among these, the CF267 × 3RXX (43.1 ± 19.8) and CJ149 × 3RXX (60.9 ± 18.9) complexes stand out as the most promising, indicating favorable predicted interactions with catalytic regions of Gram-negative pathogens, which may be relevant for future functional evaluation. Additional physicochemical parameters further reinforce these results. All docking simulations yielded negative van der Waals (*E*_vdw_), electrostatic (*E*_elec_), and desolvation (*E*_desol_) terms within the HADDOCK scoring function, reflecting favorable shape complementarity, stable physical contact, strong charge-driven attraction, and a well-formed hydrophobic interface. Specifically, CF267 showed *E*_vdw_ = −72.4, *E*_elec_ = −165.9, and *E*_desol_ = −22.9, while CJ149 exhibited *E*_vdw_ = −68.5, *E*_elec_ = −320.9, and *E*_desol_ = −7.3. Together, these values support the favorable predicted interaction profiles of both peptide-enzyme complexes.^23,24^

**Figure 5:**
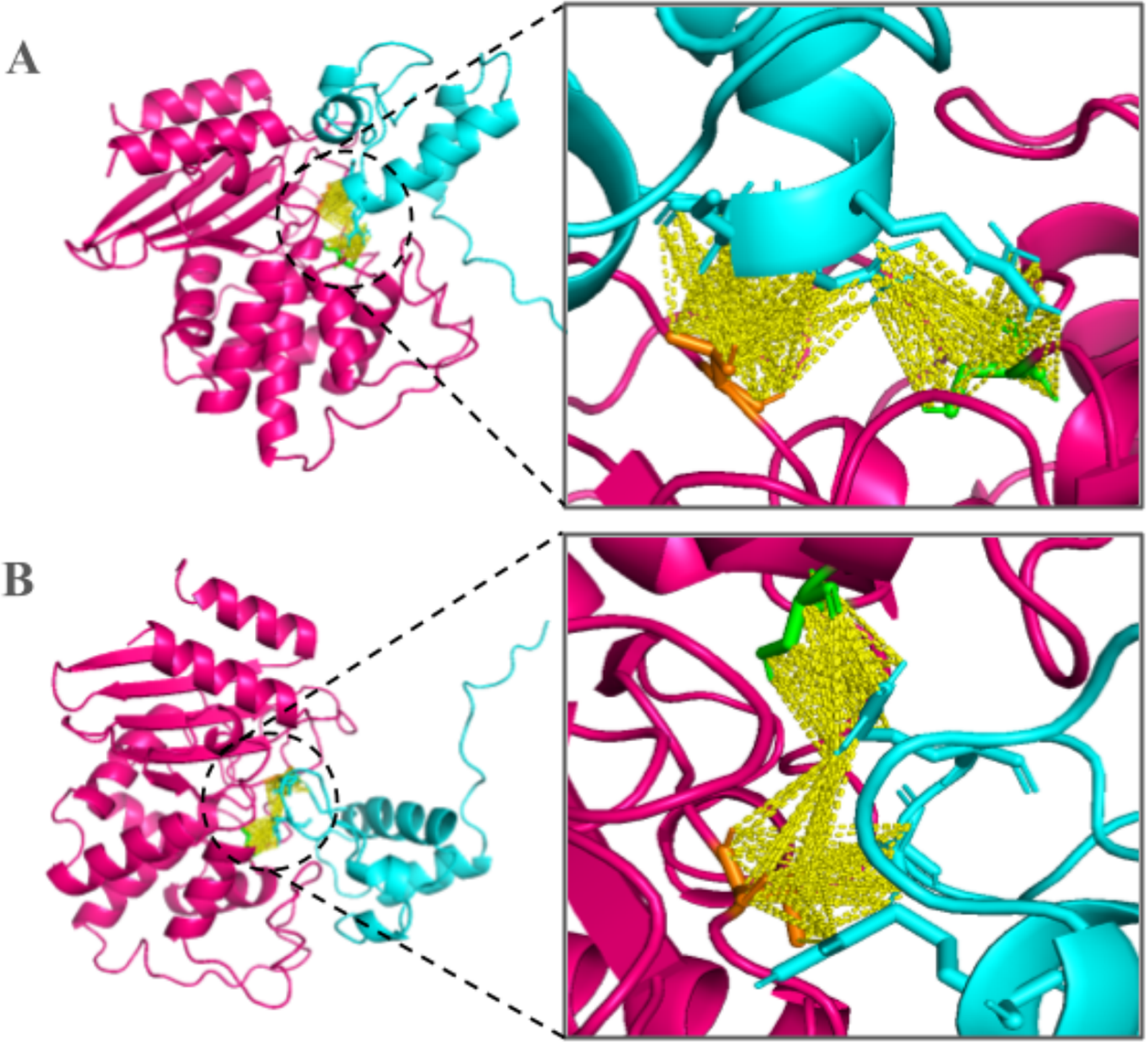
Interactions between the two key residues Ser130 (site #1, orange) and Thr237 (site #2, green) and the two AMPs. (A) CF267 forms five hydrogen bonds: three involving residues Arg47, Cys48, and Arg49 at site #1, and two involving Arg47 and Arg49 at site #2. (B) CJ149 forms four hydrogen bonds: three involving Arg32, Tyr36, and Asn68 at site #1, and one involving Asn68 at site #2.

#### Potential Rupture of Phospholipidic Bilayer Membrane

When analyzing interactions between targets and ligands, most studies focus on intracellular interactions, aiming to block specific protein functions, signaling pathways, or metabolite expression. However, one of the main characteristics of AMPs is their ability to interact directly with the outer membrane of microorganisms. This is one of the reasons why AMPs present a challenge to AMR; they do not depend on specific protein targets. To understand these potential membrane interactions, several physicochemical properties of AMPs are particularly relevant, including net charge, isoelectric point (pI), hydrophobicity, and amino acid composition.^14,37^ It is important to note that the CHARMM-GUI analysis performed in this study represents a structural modeling approach and does not include full molecular dynamics simulations of membrane disruption processes. By analyzing these features, it becomes possible to describe membrane-targeting mechanisms such as the barrel-stave, carpet, and toroidal-pore models. All of these involve interactions with the cell membrane that ultimately lead to rupture and cell lysis.^38^

The secondary structure of AMPs (whether α-helix, β-sheet, linear, or mixed) can help predict which mechanism is likely to occur (Yang et al.). In general, AMPs are cationic, carrying a positive charge that allows electrostatic attraction to the negatively charged phospholipid bilayer, leading to membrane disruption and lysis.^38^ The mechanisms of action depend on different characteristics; the most famous are defensins and cathelicidin families.^13^

In the barrel-stave model, peptides often contain β-sheet structures and assemble through interactions involving their hydrophilic regions (eg, alamethicin, pardaxin^39^). The carpet model, typically associated with α-helical peptides such as cathelicidin LL-37, magainins, temporins, and cecropins,^11,40^ is mainly established for residues like alanine (Ala), leucine (Leu), and lysine (Lys).^40^ In contrast, β-sheet structures are more commonly found in AMPs from invertebrates and plants (thionine), particularly within the defensin superfamily, whose activity depends on the specific arrangement of three to five disulfide bonds.^13,40^ The carpet model acts in a *“detergent-like”* manner, promoting peptide aggregation on the membrane surface and leading to membrane rupture.

The toroidal-pore model (e.g., aurein, melittin), observed in cationic peptides such as TC19 and TC84, involves the induction of local membrane curvature, where phospholipid head groups and peptides align cooperatively in proportional amounts, forming transient, fluidic pore-like structures. This model exhibits a dynamic and irregular organization.^37–39^ LL-37, a cathelicidin-family AMP rich in proline residues, exhibits potent activity against both Gram-negative and Gram-positive bacteria, including *E. coli*, *S. aureus*, and *P. aeruginosa*.^39^ Additionally, AMPs such as CAP18, CAP35, and lactoferrin-derived peptides can inhibit LPS-induced cytokine release from macrophages, thereby downregulating the inflammatory response.^11^

Additionally, for AMPs that act through toroidal-pore or carpet-like mechanisms, these interactions can be simulated using computational tools such as CHARMM-GUI, which allows the modeling of peptide–membrane dynamics. The simulation of the Gram-negative phospholipid bilayer was performed on the CHARMM-GUI server following the tutorial developed by the tool’s authors. In this analysis, *C. jejuni* was used as the default model organism. This membrane composition is the default CHARMM-GUI Gram-negative model and serves as a general approximation of Gram-negative outer membranes, rather than a species-specific reconstruction. Given the relatively long length of these peptide sequences, their interaction mode is compatible with the carpet mechanism, which involves surface aggregation rather than pore formation, leading to membrane disruption through a detergent-like effect.

The CF267 and CJ149 were simulated using the CHARMM-GUI platform, and in both cases, the AMPs exhibited strong interactions with the phospholipid bilayer, indicating possible membrane affinity in the simulated environment. However, such interactions predicted in silico do not necessarily translate into antimicrobial activity in biological systems and should therefore be interpreted cautiously. As illustrated in Figure 6, the bottom and top views reveal that these simulated peptides appear capable not only of interacting with the LPS layer but also to the inserting into the hydrophobic core within the computational model of the membrane, thereby suggesting possible interaction and insertion within the simulated membrane environment in the computational model, which may indicate a possible mechanism of membrane disruption but does not necessarily imply bacterial cell lysis under biological conditions. In Gram-negative bacteria, this process involves the peptides first crossing the outer phospholipid membrane, then passing through the peptidoglycan layer to finally reach the inner (cytoplasmic) membrane.^13,40^ These membrane interactions do not imply that the peptides necessarily reach the periplasmic space where β-lactamases reside, where β-lactamases are located, and additional biological constraints must be considered.

**Figure 6:**
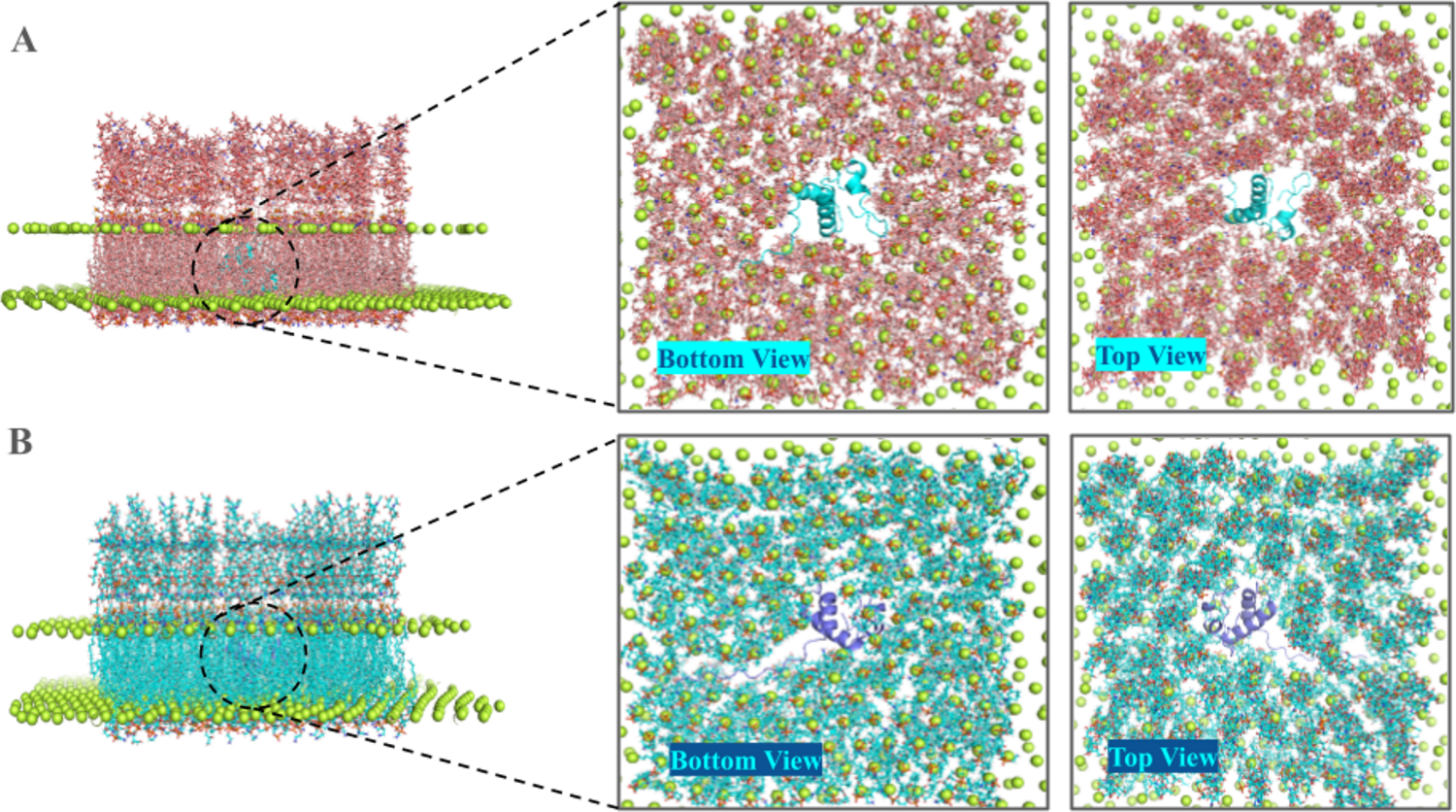
Structural representation of the phospholipid bilayer membrane of a Gram-negative bacterium (e.g., *C. jejuni*), generated using the CHARMM-GUI workflow. (A) Interaction of CF267 within the membrane, shown from both bottom and top views. (B) Interaction of CJ149 within the membrane, shown from both bottom and top views. Both AMPs demonstrate potential interactions with the pathogen membrane, which may indicate membrane affinity and potential interaction with the bilayer membrane in Gram-negative bacteria.

An important biological consideration concerns the cellular localization of β-lactamases in Gram-negative bacteria. These enzymes are typically located in the periplasmic space, which is separated from the extracellular environment by the outer membrane.^13,40^ Therefore, although the docking results suggest potential compatibility between the peptides and β-lactamase binding regions, it remains unclear whether peptides of this size and physicochemical nature can efficiently cross the outer membrane and reach the periplasmic compartment.^13,40^ This limitation highlights that the proposed enzyme-targeting mechanism should be interpreted cautiously. The observed interactions may instead reflect structural compatibility rather than effective inhibition in vivo. Alternatively, peptide activity may be primarily associated with membrane interactions or other extracellular effects, with β-lactamase binding representing a secondary or conditional mechanism that would depend on peptide uptake and intracellular access.

The docking results instead illustrate potential biochemical compatibility, which requires experimental validation regarding cellular uptake and periplasmic access. In addition, important bacterial defense mechanisms should be considered when interpreting the biological relevance of the predicted interactions. Gram-negative pathogens such as *K. pneumoniae* and *A. baumannii* possess protective features, including polysaccharide capsules and multidrug efflux systems that may reduce peptide penetration or promote peptide extrusion from the periplasmic space. These factors may limit the effective concentration of antimicrobial peptides at the site where β-lactamases are located, highlighting the importance of future experimental validation and bacterial uptake studies.

## Conclusion

This study highlighted the importance of identifying potential interaction regions, binding pockets, and catalytic sites in pathogen-derived β-lactamases, key enzymes associated with the clinical emergence of AMR. As a promising therapeutic alternative, AMPs were investigated for their predicted interactions with enzyme binding regions and membrane models, aiming to explore their potential relevance for antimicrobial applications. The results indicated that all five *Cereus*-derived AMPs were capable of forming stable *in silico* complexes with β-lactamase interaction sites and of engaging the phospholipid bilayer in the simulated system, suggesting potential membrane affinity rather than confirmed disruption. Moreover, docking analyses revealed favorable binding scores for all peptides against both β-lactamase targets. Among them, CF267 × 3RXX (43.1 ± 19.8; *E*_vdw_ = −72.4, *E*_elec_ = −165.9, *E*_desol_ = −22.9) and the CJ149 × 3RXX (60.9 ± 18.9; *E*_vdw_ = −68.5, *E*_elec_ = −320.9, *E*_desol_ = −7.3) emerged as the most promising complexes, indicating favorable predicted binding interactions with β-lactamase targets and suggesting a potential inhibitory mechanism that requires experimental validation. These predictions, however, do not provide direct evidence of enzymatic inhibition and require experimental validation through biochemical and microbiological assays. Thus, the next steps will involve *in vitro* assays to validate these findings and confirm the antimicrobial and enzyme–inhibitory activity of the selected peptides.

## Supplementary Material



## Notes

Main features of the probes used in FTSite and FTMap, including functional group, polarity, hydrogen-bonding potential, and molecular structures; PDB IDs of β-lactamase structures and their corresponding predicted binding pockets, including pocket score and druggability values; and association between β-lactamase structures and predicted potential pocket regions (PDF)

## Abbreviations

ABPs: antibacterial proteins
AFPs: antifungal proteins
AMPs: antimicrobial peptides
AMR: antimicrobial resistance
MDR: multidrug-resistant
CSs: consensus sites
LPS: lipopolysaccharides
PDB: protein data bank
