# Correction to “Bioinformatics Analysis of *Cereus*-Derived Peptides Targeting β‑Lactamases and Bilayer Membrane from *Klebsiella pneumoniae* and *Acinetobacter baumannii*”

**DOI:** 10.1021/acsomega.6c08696

**Published:** 2026-08-29

**Authors:** João A. Teodoro, Maria Izadora O. Cardoso, Graziela S. Virgens, Danilo T. Amaral

In the published version of the article,^1^ Table 3 contains incorrect punctuation/formatting in the numerical values. The correct version of Table 3 is given below. This correction is limited to formatting and does not affect the results, data interpretation, or conclusions of the study.

**Table 3:** Computed Structural Features of the Peptide Models

**Peptide**	**SASA Total**	**Fraction Hydrophobic Surface**	**RG**	**Disulfide Pairs**	**PLDDT Mean**	**PLDDT Median**	**Fraction PLDDT LT70**	**Contact Density**
CF15	6437.44	0.19	14.26	4	89.62	96.94	0.13	8.81
CF267	6385.25	0.26	16.83	5	90.45	96.91	0.17	7.97
CH167	6102.94	0.28	14.05	5	86.41	93.94	0.19	8.08
CH176	4948.63	0.29	11.74	3	95.84	96.44	0.0	9.0
CJ149	5684.45	0.25	13.38	5	85.89	93.44	0.17	8.62
