# Supplementary material for: Bioinformatics Analysis of Cereus-Derived Peptides Targeting β‑Lactamases and Bilayer Membrane from Klebsiella pneumoniae and Acinetobacter baumannii
Source: ACS Omega. 2026 Jun 2;11(23):33630–41. doi: 10.1021/acsomega.5c13072 (PMC13280821; doi:10.1021/acsomega.5c13072)
Supplement: Supplementary file 1 [file ao5c13072_si_001.pdf]

Bioinformatics Analysis of *Cereus*-Derived Peptides targeting β-Lactamases and Bilayer Membrane from *Klebsiella pneumoniae* and *Acinetobacter baumannii*

João A. Teodoro[1*], *Maria Izadora O. Cardoso*[1], *Graziela S. Virgens*[1], *and Danilo T. Amaral*[1]

Centro de Ciências Naturais e Humanas, Universidade Federal do ABC (UFABC), Santo André, São Paulo, Brasil.

Table S1. Main features of the probes used in FTSite and FTMap, including molecule name, functional group, polarity, hydrogen-bonding potential, and molecular structure. All information was obtained from PubChem (https://pubchem.ncbi.nlm.nih.gov/) and organized to facilitate understanding.

Molecule	Functional Group	Polarity	H-binding capacity	Structure
Acetamide	Amide	Polar	Donor and acceptor	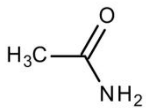
Acetonitrile	Nitrile	Polar	Acceptor	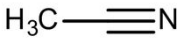

Acetone	Ketone	Polar	Acceptor	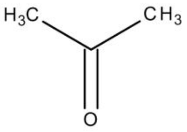
Acetaldehyde	Aldehyde	Polar	Acceptor	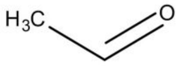
Methylamine	Primary amine	Polar	Donor and acceptor	H_2N-CH_3
Benzaldehyde	Aromatic aldehyde	Polar	Acceptor	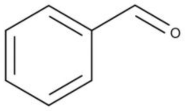
Benzene	Aromatic hydrocarbon	Nonpolar	None	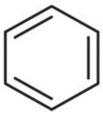
Isobutanol	Alcohol	Polar	Donor and acceptor	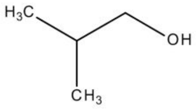
Cyclohexane	Alkane	Nonpolar	None	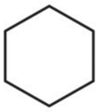
N,N-dimethylformamide	Tertiary amide	Polar	Acceptor	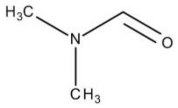
Dimethyl ether	Ether	Slightly polar	Acceptor	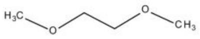
Ethanol	Alcohol	Polar	Donor and acceptor	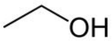
Ethane	Alkane	Nonpolar	None	H_3C-CH_3
Phenol	Aromatic alcohol	Polar	Donor and acceptor	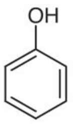
Isopropanol	Secondary alcohol	Polar	Donor and acceptor	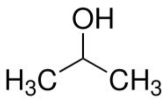

Urea	Amide	Polar	Donor and acceptor	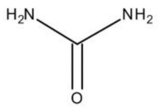

Table S2. PDB IDs of the two β-lactamase types and their corresponding binding pockets, including pocket score, druggability value, pocket color, and the associated pathogen protein.

B-lactamase	Pockets	Score	Druggability	Color
3RXX	1	0.286	0.60	wheat
3RXX	2	0.240	0.003	palegreen
3RXX	3	0.201	0.005	lightblue
3RXX	4	0.161	0.05	lightpink
3RXX	5	0.144	0.424	paleyellow
3RXX	6	0.063	0.000	palecyan
3RXX	7	0.062	0.006	lightorange
3RXX	8	0.051	0.004	bluewhite
3RXX	9	0.028	0.004	purpleblue
3RXX	10	0.220	0.001	gray
3RXX	11	0.017	0.000	salmon
3RXX	12	0.010	0.001	limegreen
3RXX	13	0.004	0.000	orange
3RXX	14	-0.031	0.000	marine
4U0T	1	0.468	0.855	wheat
4U0T	2	0.319	0.377	palegreen
4U0T	3	0.204	0.016	lightblue
4U0T	4	0.187	0.020	lightpink
4U0T	5	0.184	0.031	paleyellow
4U0T	6	0.180	0.541	palecyan
4U0T	7	0.111	0.000	lightorange
4U0T	8	0.109	0.005	bluewhite
4U0T	9	0.108	0.003	purpleblue

4U0T	10	0.097	0.003	gray
4U0T	11	0.091	0.002	salmon
4U0T	12	0.085	0.001	limegreen
4U0T	13	0.081	0.001	orange
4U0T	14	0.079	0.004	marine
4U0T	15	0.060	0.001	brown
4U0T	16	0.055	0.006	smudge
4U0T	17	0.049	0.000	blue
4U0T	18	0.033	0.040	yellow
4U0T	19	-0.035	0.02	raspberry

Table S3. Association between the β-lactamase structures and the structural sites that correspond to the predicted potential pocket regions.

B-lactamase	Site	Pocket
3RXX	1	2, 4, and 9
3RXX	2	5
3RXX	3	1
4U0T	1	4, 7, and 10
4U0T	2	10
4U0T	3	6 and 17
